# Reciprocal Regulation Between Smad7 and Sirt1 in the Gut

**DOI:** 10.3389/fimmu.2018.01854

**Published:** 2018-08-10

**Authors:** Silvia Sedda, Eleonora Franzè, Gerolamo Bevivino, Martina Di Giovangiulio, Angelamaria Rizzo, Alfredo Colantoni, Angela Ortenzi, Enrico Grasso, Mario Giannelli, Giuseppe S. Sica, Massimo Claudio Fantini, Giovanni Monteleone

**Affiliations:** ^1^Department of Systems Medicine, University of Rome Tor Vergata, Rome, Italy; ^2^Department of Surgery, University of Rome Tor Vergata, Rome, Italy

**Keywords:** mucosal inflammation, colitis, transforming growth factor-β, Sirt1, inflammatory bowel diseases

## Abstract

In inflammatory bowel disease (IBD) mucosa, there is over-expression of Smad7, an intracellular inhibitor of the suppressive cytokine transforming growth factor-β1, due to post-transcriptional mechanisms that enhance Smad7 acetylation status thus preventing ubiquitination-mediated proteosomal degradation of the protein. IBD-related inflammation is also marked by defective expression of Sirt1, a class III NAD+-dependent deacetylase, which promotes ubiquitination-mediated proteosomal degradation of various intracellular proteins and triggers anti-inflammatory signals. The aim of our study was to determine whether, in IBD, there is a reciprocal regulation between Smad7 and Sirt1. Smad7 and Sirt1 were examined in mucosal samples of IBD patients and normal controls by Western blotting and immunohistochemistry, and Sirt1 activity was assessed by a fluorimetric assay. To determine whether Smad7 is regulated by Sirt1, normal or IBD lamina propria mononuclear cells (LPMC) were cultured with either Sirt1 inhibitor (Ex527) or activator (Cay10591), respectively. To determine whether Smad7 controls Sirt1 expression, *ex vivo* organ cultures of IBD mucosal explants were treated with Smad7 sense or antisense oligonucleotide. Moreover, Sirt1 expression was evaluated in LPMC isolated from Smad7-transgenic mice given dextran sulfate sodium (DSS). Upregulation of Smad7 was seen in both the epithelial and lamina propria compartments of IBD patients and this associated with reduced expression and activity of Sirt1. Activation of Sirt1 in IBD LPMC with Cay10591 reduced acetylation and enhanced ubiquitination-driven proteasomal-mediated degradation of Smad7, while inhibition of Sirt1 activation in normal LPMC with Ex527 increased Smad7 expression. Knockdown of Smad7 in IBD mucosal explants enhanced Sirt1 expression, thus suggesting a negative effect of Smad7 on Sirt1 induction. Consistently, mucosal T cells of Smad7-transgenic mice contained reduced levels of Sirt1, a defect that was amplified by induction of DSS colitis. The data suggest the existence of a reciprocal regulatory mechanism between Smad7 and Sirt1, which could contribute to amplify inflammatory signals in the gut.

## Introduction

Inflammatory bowel diseases (IBD) are chronic inflammatory disorders of the gastrointestinal tract with a multifactorial pathophysiology. Accumulating evidence suggests that both Crohn’s disease (CD) and ulcerative colitis (UC), the two major IBD, arise because of the action of multiple environmental and genetic factors, which ultimately promote an excessive immune response against component of the luminal flora (1, 2). Defects in counter-regulatory mechanisms are also documentable in inflamed tissue of IBD patients and supposed to amplify the pathological process. For instance, in both IBD, there is diminished activity of transforming growth factor (TGF)-β1, an immunosuppressive cytokine that delivers negative signals in many immune cells (3). Such a defect has been associated with elevated levels of Smad7, an intracellular protein that binds to TGF-β receptor type I and inhibits TGF-β1-induced signaling (4, 5). Consistently, inhibition of Smad7 with a specific antisense oligonucleotide (AS) restores TGF-β1 signaling and reduces inflammatory pathways in both *in vitro* and *in vivo* models of intestinal inflammation (5, 6).

The mechanisms/factors regulating Smad7 expression in IBD mucosa are not fully understood. Our previous studies showed that Smad7 is not regulated at transcriptional level as no significant change in Smad7 RNA expression was found between IBD and normal control samples (7). It was also shown that, in IBD, high Smad7 is sustained by post-transcriptional mechanisms that enhance its stability (7). Indeed, Smad7 is highly acetylated on lysine residues and this protein modification prevents ubiquitination-driven proteasomal-mediated degradation of Smad7 (8). Analysis of factors involved in the control of Smad7 acetylation revealed that IBD-associated inflammation is marked by elevated levels of p300, a protein with intrinsic acetyltransferase activity (7). Silencing of p300 with a specific siRNA partially reduced Smad7 content (7), suggesting the existence of further mechanisms/factors involved in the control of Smad7 protein stability. One such factor could be Sirt1, a class III histone deacetylase, which enhances ubiquitination-driven proteasome-mediated degradation of various inflammatory proteins (9). Notably, Sirt1 expression is reduced in IBD tissue and activation of Sirt1 attenuates inflammatory signals in the gut, raising the possibility that such a defect could contribute to perpetuate pathogenic responses in the gut (10). In an attempt to dissect the mechanisms that suppress Sirt1 in IBD, we showed that stimulation of control intestinal mucosal cells with tumor necrosis factor (TNF)-α and interleukin (IL)-21 reduced Sirt1 expression while blockade of the activity of these two cytokines upregulated Sirt1 in IBD lamina propria mononuclear cells (LPMC) (10). This suggests that reduction of Sirt1 in IBD can rely on factors generated within the inflammatory microenvironment. Based on these findings, we hypothesized that, in IBD mucosa, reduced Sirt1 activity contributes to enhance Smad7 protein stability and expression, while high Smad7 helps propagate signals that culminate in Sirt1 downregulation. Therefore, the aim of this study was to investigate whether, in IBD, there is a reciprocal regulatory mechanism between Smad7 and Sirt1.

## Materials and Methods

### Patients and Samples

Mucosal biopsies were taken from the inflamed areas of six patients with CD (three females and three males) and six patients with UC (two females and four males) (age ranging from 18 to 60 years) undergoing colonoscopy for a clinically active disease at the Gastroenterology Unit of Tor Vergata University Hospital (Rome, Italy). In three of six CD patients, primary site of involvement was the colon while the remaining patients had an ileal disease. Four CD patients were receiving mesalazine, one patient was receiving steroids and mesalazine, and one patient was receiving sulfasalazine. All patients had a CD activity index >150. Three of six UC patients had a left-sided colitis and three had an extensive colitis; all the patients had endoscopic evidence of active disease (Mayo endoscopic score ≥2) at the time of enrollment. Five UC patients were receiving mesalazine, and one patient was receiving sulfasalazine. Controls included biopsies taken from unaffected colonic mucosa of six subjects undergoing colonoscopy for colorectal cancer screening. Surgical specimens were taken from eight patients with CD (two females and six males) and six patients with UC (two females and four males) undergoing surgery for a chronic active disease poorly responsive to medical treatment with steroids, immunosuppressive, or biological drugs. At the time of surgery, six CD patients had a stricturing disease and two had a fistulizing disease. All UC patients had an extensive colitis and were receiving corticosteroids. Controls included mucosal specimens taken from macroscopically and microscopically unaffected colonic areas of eight patients undergoing surgery for colon cancer. Surgical specimens were used for protein extraction and LPMC isolation.

Each patient who took part in the study gave written informed consent. The study was approved by the Independent Ethic Committee at the Policlinico Tor Vergata of Rome (Rome, Italy) (experimentation register no. 42/12).

### LPMC Isolation

All reagents were from Sigma-Aldrich unless specified. LPMC were isolated from mucosal specimens as previously described (11). LPMC were re-suspended in X-Vivo supplemented with penicillin (P) (100 µg/ml), streptomycin (S) (100 µg/ml), and gentamycin (50 µg/ml, Lonza, Verviers, Belgium). Control LPMC were pre-incubated with Ex527 (100 µM, Tocris, Bristol, UK) or dimethyl sulfoxide (DMSO) (vehicle), whereas IBD LPMC were incubated with Cay10591 (10 µM, Cayman chemical) or DMSO for 8 h at 37°C. In additional experiments, IBD LPMC were pre-incubated with proteasome inhibitors (Mg112 and Mg132, final concentration 25 µM, Calbiochem-Merck Darmstadt, Germany) for 24 h and then treated with Cay10591 or DMSO for eight further hours.

### Organ Cultures

Mucosal biopsy samples of IBD patients were placed on transwell (transwell permeable support, Costar, Corning incorporated, New York, NY, USA) in 24-well plates containing X-VIVO medium supplemented with 1% P/S and 50 µg/ml gentamycin in the presence of either Smad7 sense oligonucleotide or AS (both used at 10 µg/ml) (5). The culture was performed in an organ culture chamber at 37°C in a 5% CO_2_/95% O_2_ atmosphere and after 24 h the samples were used for protein extraction.

### Immunohistochemistry

Paraffin sections of specimens of CD patients, UC patients, and healthy controls were incubated with anti-human Smad7 mouse monoclonal antibody (R&D Systems, Minneapolis, MN, USA, 1:50 final dilution) or anti-human Sirt1 mouse monoclonal antibody (Millipore, Milan, Italy, 1:100 final dilution). Isotype control IgG-stained sections were prepared under identical immunohistochemical conditions, replacing the primary antibody with a purified mouse IgG control antibody (R&D Systems). Positive cells, in both the epithelial and lamina propria compartments, were counted in four different fields per section.

### Total Protein Extraction, Western Blotting, and Sirt1 Activity Assay

Lamina propria mononuclear cells were lysed on ice with a buffer containing 10 mM HEPES (pH 7.9), 10 mM KCl, 0.1 mM EDTA, and 0.5% Nonidet p40, supplemented with 1 mM dithiothreitol, 10 mg/ml aprotinin, 10 mg/ml leupeptin, 1 mM phenyl-methylsulfonyl fluoride, 1 mM Na_3_VO_4_, and 1 mM NaF. Additional samples, which were lysed with the above buffer without protease inhibitors, were used for assessing Sirt1 activity. Lysates were clarified by centrifugation at 12,000 *g* for 30 min at 4°C and separated on 10% sodium dodecyl sulfate (SDS)-polyacrylamide gel electrophoresis. Smad7 was detected using a monoclonal mouse anti-human antibody (R&D Systems, final concentration 1 µg/ml) followed by a horseradish peroxidase–conjugated rabbit anti-mouse IgG monoclonal antibody (Dako, Milan, Italy). The reactions were detected with a sensitive enhanced chemiluminescence kit (Pierce, Rockford, IL, USA). Computer-assisted scanning densitometry (Chemidoc Touch Images; Bio-Rad) was used to analyze the intensity of the immunoreactive bands. After the analysis, blots were stripped and incubated with a mouse anti-human β-actin antibody as internal loading control, followed by a rabbit anti-mouse antibody conjugated to horseradish peroxidase (Dako) to ascertain equivalent loading of the lanes.

The enzymatic activity of Sirt1 was assessed using the Sirt1 deacetylase fluorimetric assay kit according to the manufacturer’s instructions (Abnova, Taipei, Taiwan).

### Immunoprecipitation

For immunoprecipitation, the protein lysates were prepared from control LPMC treated with Ex527 or DMSO for 8 h and IBD LPMC treated with Cay10591 or DMSO. Proteins were immunoprecipitated with 2 μg/500 μl of anti-Smad7 (Santa Cruz Biotechnology, Santa Cruz, CA, USA) or control isotype antiserum for 2 h followed by incubation with protein A/G agarose beads overnight. The resulting immunoprecipitates were washed four times with cold lysis buffer, separated by SDS-polyacrylamide gel electrophoresis, and immunoblotted with antibodies against acetyl-lysine (final dilution 1:2,000, Cell Signaling Technology, Danvers, MA, USA) followed by a horseradish peroxidase-conjugated anti-rabbit IgG antibody (Promega, Madison, WI USA) or with antibody against ubiquitin (final dilution 1:400, Santa Cruz Biotechnology) followed by a horseradish peroxidase-conjugated anti-mouse IgG antibody. The reactions were detected and analyzed as above. At the end, the blots were stripped and incubated with anti-Smad7 antibody (final concentration 1 µg/ml, R&D Systems).

### Experimental Colitis and LPMC Isolation

For induction of colitis, dextran sulfate sodium (DSS; MW, 36,000–50,000 Da; MP Biomedicals, Solon OH, USA) was given in the drinking water for 7 days to 11-week-old female C57BL/6 wild-type (WT) or CD2-Smad7 Transgenic (Tg) mice generated as elsewhere reported (12). Mice were kept on water for 3 days after the DSS treatment. Controls consisted of C57BL/6 mice receiving drinking water without DSS. Mice were daily monitored for the development of clinical signs of colitis (e.g., body weight changes) and killed at day 10. At the end of the experiments, colons were removed and collected for histology, RNA extraction, and LPMC isolation. Colonic sections were obtained for hematoxylin and eosin staining and analyzed by light microscope. The colitis score was assigned as described elsewhere (13). The colons were opened longitudinally and washed with Ca^2+^ and Mg^2+^ free HBSS and LPMC were isolated using the Lamina Propria Dissociation Kit mouse protocol (Miltenyi Biotech, Bergisch-Gladbach, Germany).

The study was approved by the Ministry of Health (authorization no. 324/2016-PR).

### Flow Cytometry Analysis

Murine LPMC were washed in PBS and stained with the following surface antibodies: Pacific Blue-labeled against CD3 (clone 500A2), APC-Cy7-labeled against CD4 (clone GK1.5) (BD Pharmingen, Milan, Italy), and Pe-Cy7-labeled antibodies against CD11b (MI/70), CD11c (HL3), and CD19 (1D2) (all BD Pharmingen) for 15 min at room temperature (RT). Intracellular staining was performed using Alexa 488 (FITC)-labeled antibody against Sirt1 (19A7AB4) (Abcam, Cambridge, UK) for 30 min at RT. Appropriate isotype-matched control IgG (BD Biosciences) were included in all the experiments. Sirt1-positive cells were analyzed by flow cytometry (FACS VERSE; BD Bioscience, San Jose, CA, USA) gating on living cells according to LIVE/DEAD^®^ staining (Thermo Fisher Scientific).

### RNA Extraction, Complementary DNA (cDNA) Preparation, and Real-Time PCR

Total RNA was extracted from colonic sections of WT and Smad7 Tg mice using Pure Link mRNA mini kit according to the manufacturer’s instructions (Life Technologies, Milan, Italy). A constant amount of RNA (1 mg/sample) was retro-transcribed into cDNA and then 1 ml of cDNA per sample was amplified using a SYBER Green master mix (Bio-Rad, Milan, Italy). PCR was performed using the following conditions: denaturation 1 min at 95°C; annealing 30 s at 58°C for mTNF-α and mLCN2; at 60°C for β-actin, mIL-6, and mIFN-γ, followed by 30 s of extension at 72°C. Primers sequence was as follows: mTNF-α forward 5′-ACCCTCACACTCAGATCATC-3′, reverse 5′-GAGTAGACAAGGTACAACCC-3′; mLCN2 forward 5′-CCAGGACTCAACTCAGAAC-3′, reverse 5′-GCTCATAGATGGTGCTGTAC-3′; mIL-6 forward 5′-CCATAGCTACCTGGAGTACATG-3′, reverse 5′-TGGAAATTGGGGTAGGAAGGAC-3′; mIFN-γ forward 5′-TCAAGTGGCATAGATGTGGAAGAA-3′, reverse 5′-TGGCTCTGCAGGATTTTCATG-3′; β-actin forward 5′-AAGATGACCCAGATCATGTTTGAGACC-3′, reverse 5′-AGCCAGTCCAGACGCAGGAT-3′, was used as internal control. RNA expression was calculated relative to the housekeeping β-actin gene on the base of the ΔΔCt algorithm.

### Data Analysis

Differences between groups were compared using the Mann–Whitney *U* test, the paired, or the unpaired *t* test. Statistical differences were assessed with the GraphPad Prism statistical PC program (GraphPad Software, San Diego, CA, USA). A *p* value of less than 0.05 was considered statistically significant.

## Results

### In IBD, High Smad7 Associates With Reduced Sirt1 Activity

**Figure 1:**
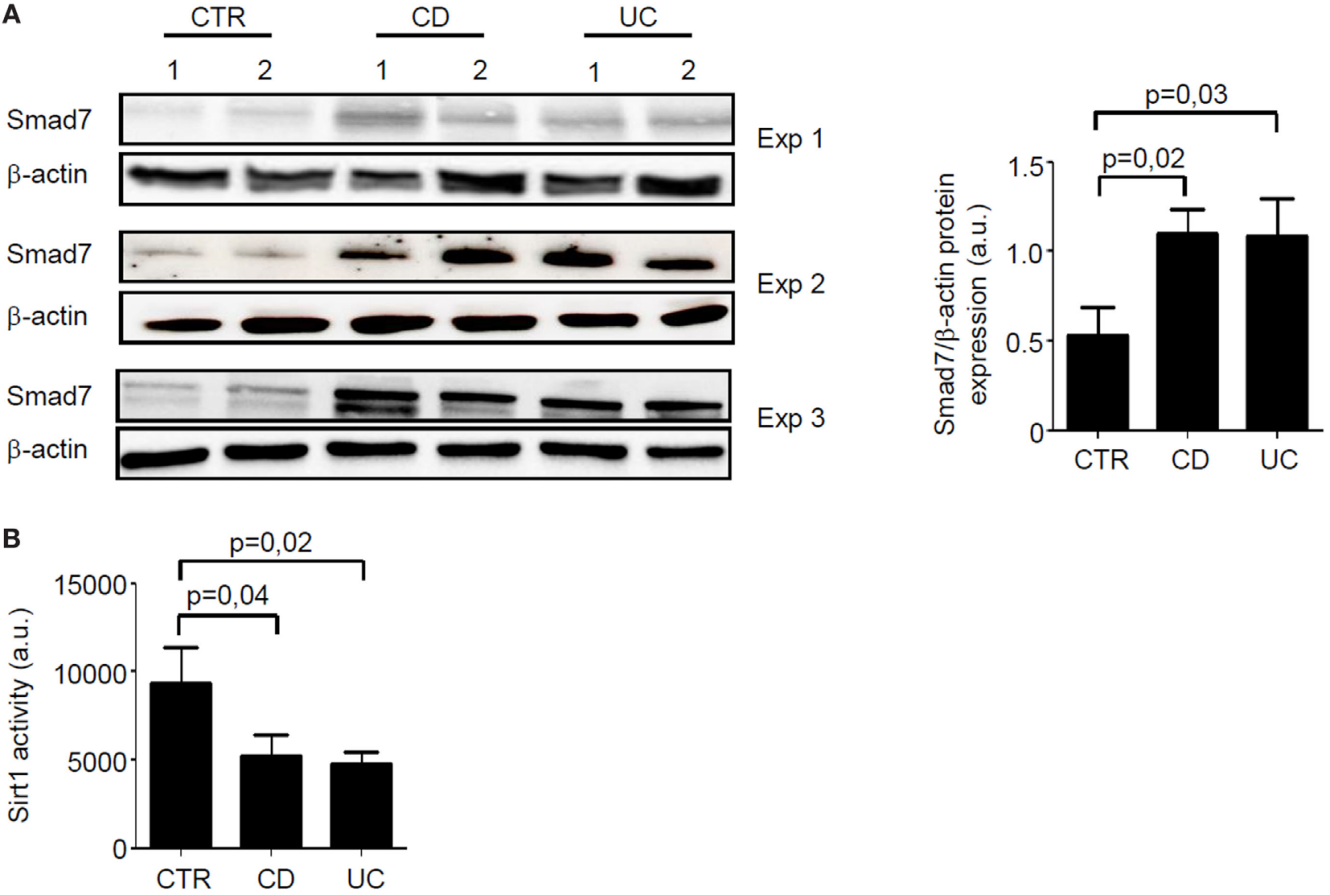
In inflammatory bowel disease, increased expression of Smad7 associates with reduced activity of Sirt1. **(A)** Western blots of three separate experiments (Exp) showing Smad7 and β-actin in total proteins extracted from surgical intestinal specimens of six healthy controls (CTR), six patients with Crohn’s disease (CD), and six patients with ulcerative colitis (UC). Right panel shows the quantitative analysis of Smad7/β-actin ratio in samples taken from six CTR, six patients with CD, and six patients with UC as measured by densitometry scanning of Western blots. Values are expressed in arbitrary units (a.u.) and indicate mean ± SEM of all the samples. **(B)** Sirt1 activity was measured in paired samples taken from the same six CTR, six patients with CD, and six patients with UC and used for Smad7 evaluation. Values are expressed in a.u. Data indicate mean ± SEM of all the samples.

Total proteins extracted from paired IBD and control samples were analyzed for Smad7 expression by Western blotting and Sirt1 activity by a fluorimetric assay. In line with our previous data (5), Smad7 expression was more pronounced in both CD and UC samples than in controls (Figure 1A). By contrast, Sirt1 activity was significantly reduced in mucosal samples of IBD patients when compared with controls (Figure 1B).

**Figure 2:**
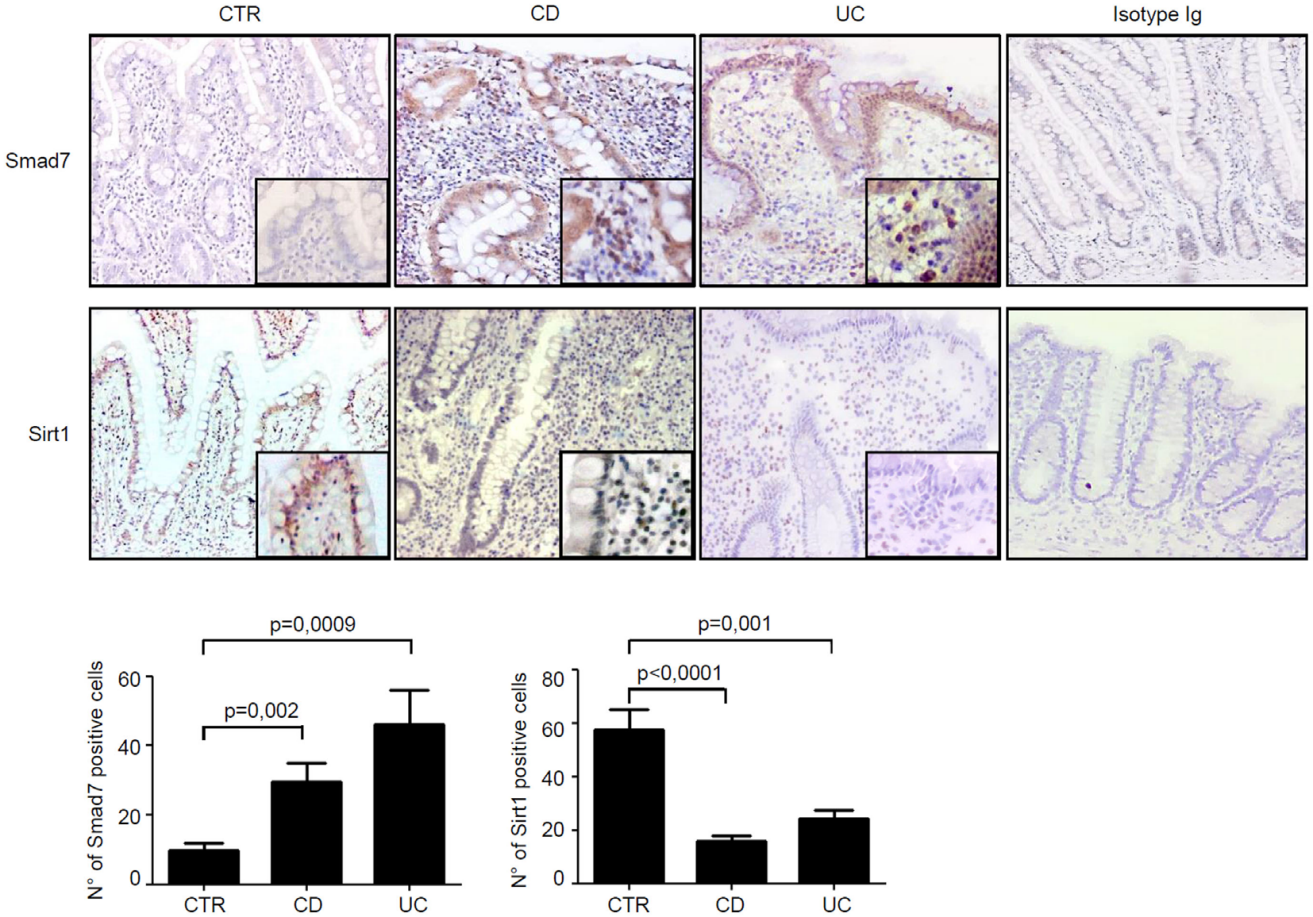
In inflammatory bowel disease, the increased expression of Smad7 associates with reduced expression of Sirt1 in both epithelial and lamina propria compartments. Representative photomicrographs (100×, original magnification) of Smad7 and Sirt1-stained paraffin-embedded sections of biopsy samples taken from one control (CTR), one patient with Crohn’s disease (CD), and one patient with ulcerative colitis (UC). Sirt1-positive cells are evident in both the epithelial and lamina propria compartments in CTR, while Smad7-positive cells are more abundant in both the epithelial and lamina propria compartments of CD patients and UC patients. Higher magnification photomicrograph (200×) is shown in the insert. Staining with control IgG is also shown. The Smad7 and Sirt1-positive cells were manually counted in at least four fields per each section taken from six CTR, six patients with CD, and six patients with UC. Data indicate mean ± SEM.

Next, we assessed the cell sources of Smad7 and Sirt1 in the gut by immunohistochemistry using sections of the same IBD patients and controls. Smad7-positive cells were more abundant in both the epithelial and lamina propria compartments of patients with IBD than in the respective compartments of controls. In control samples, Sirt1 staining was evident in both epithelial cells and LPMC, while it was very faint in IBD samples (Figure 2).

### Activation of Sirt1 Leads to Smad7 Reduction

**Figure 3:**
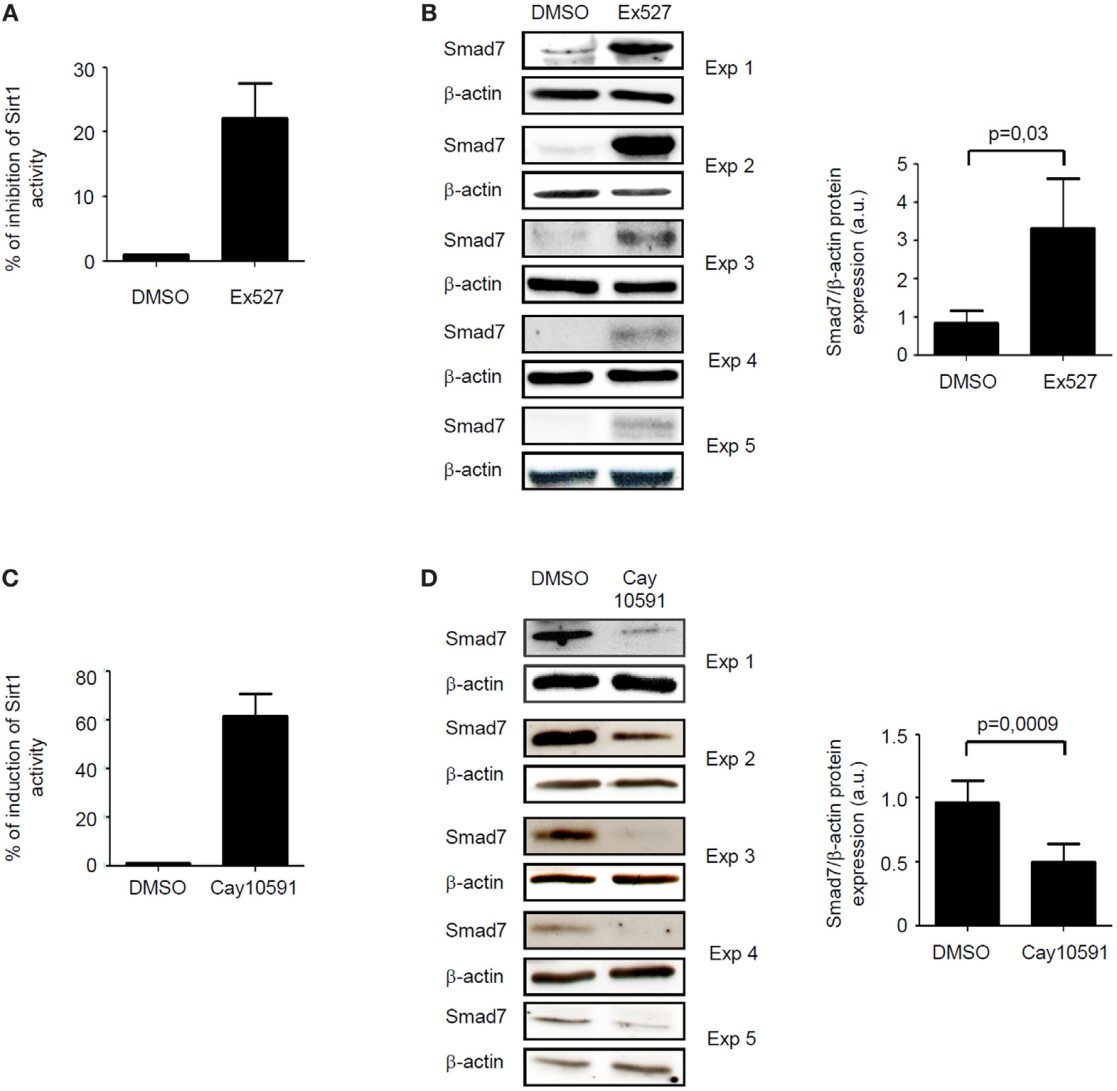
Sirt1 regulates negatively Smad7 expression. **(A)** Sirt1 activity was measured in lamina propria mononuclear cells (LPMC) isolated from five normal controls and cultured in the presence of Ex527 or dimethyl sulfoxide (DMSO). Values are expressed as percentage of inhibition of Sirt1 activity in comparison to DMSO-exposed cells. **(B)** Western blots showing Smad7 and β-actin in total proteins extracted from control LPMC and cultured in the presence of Ex527 or DMSO. Results of each experiment (Exp) are shown. Right panel shows the quantitative analysis of Smad7/β-actin ratio as measured by densitometry scanning of Western blots. Values are expressed in arbitrary units (a.u.) and indicate mean ± SEM of all samples. **(C)** Sirt1 activity was measured in LPMC isolated from five inflammatory bowel disease (IBD) patients and cultured in the presence of CAY10591 or DMSO. Values are expressed as percentage of induction of Sirt1 activity in comparison to DMSO-exposed cells. **(D)** Western blots showing Smad7 and β-actin in total proteins extracted from IBD LPMC and cultured in the presence of CAY10591 or DMSO. Results of each experiment are shown. Right panel shows the quantitative analysis of Smad7/β-actin ratio as measured by densitometry scanning of Western blots. Values are expressed in arbitrary units (a.u.) and indicate mean ± SEM of all samples.

The fact that, in inflamed tissue of IBD, high Smad7 associates with reduced activity of Sirt1, an enzyme that promotes protein degradation (9), prompted us to explore the possibility that Sirt1 is a negative regulator of Smad7 in immune cells. To this end, we treated control intestinal LPMC with Ex527, a Sirt1 inhibitor. Ex527 reduced Sirt1 activity and this associated with enhanced induction of Smad7 (Figures 3A,B). To further confirm the negative effect of Sirt1 on Smad7, IBD LPMC were treated with Cay10591, a Sirt1 activator. Activation of Sirt1 in such cells significantly reduced Smad7 expression (Figures 3C,D).

### Sirt1 Regulates the Acetylation/Ubiquitination Status of Smad7

**Figure 4:**
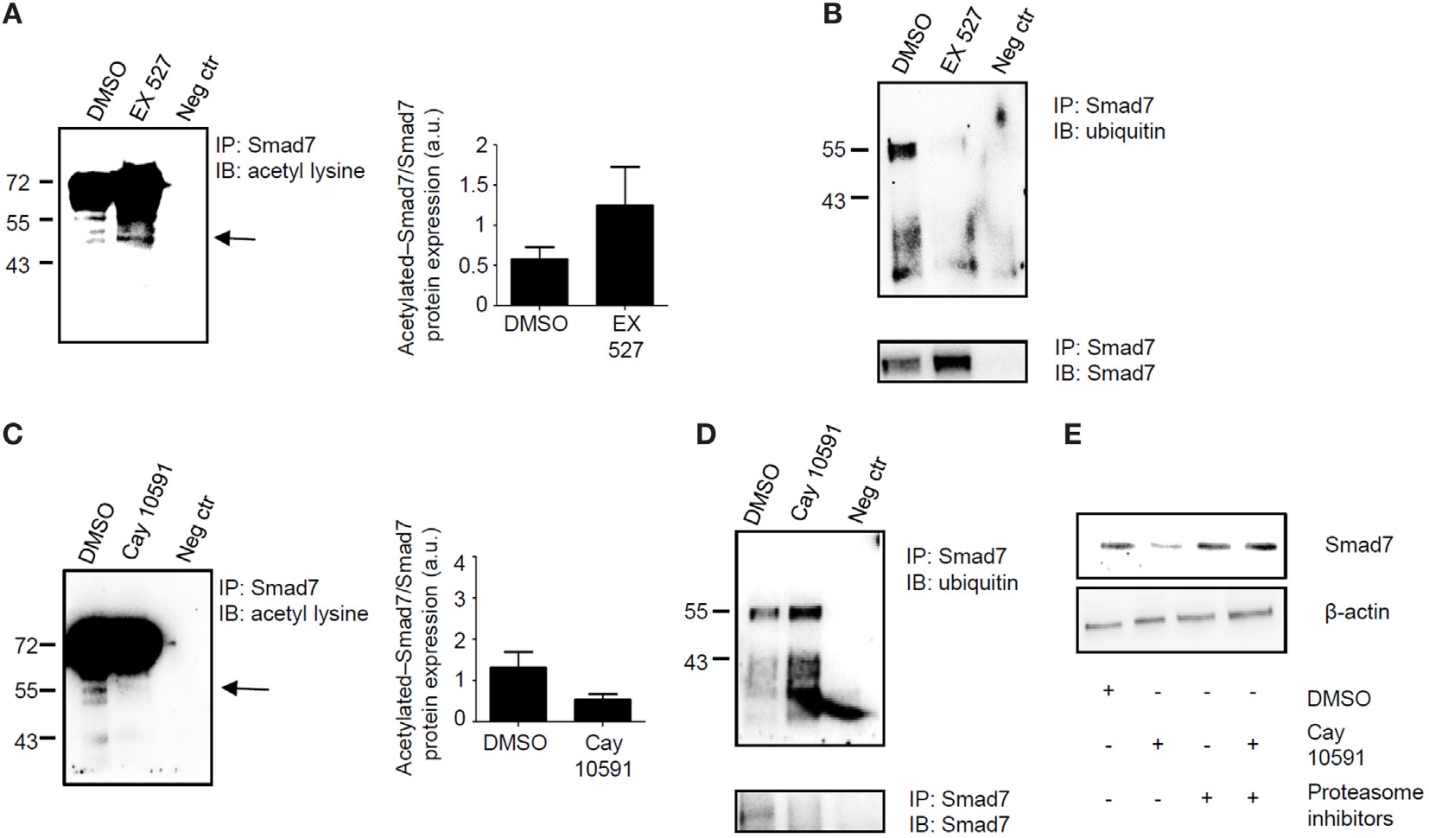
Sirt1 regulates the acetylation/ubiquitination status of Smad7. **(A,B)** Inhibition of Sirt1 in control lamina propria mononuclear cells (LPMC) increases Smad7 acetylation and reduces Smad7 ubiquitination. Total proteins extracted from control LPMC, cultured in the presence of Ex527 or dimethyl sulfoxide (DMSO) (vehicle), were immunoprecipitatated (IP) with Smad7 antibody or control antibody (neg ctr) and the blots were incubated with anti-acetyl-lysine antibody **(A)** or anti-ubiquitin antibody **(B)**. At the end, the blots were stripped and incubated with a Smad7 antibody recognizing a different epitope of that recognized by the antibody used for immunoprecipitation In panel **(A)**, arrow shows the specific band corresponding to acetylated Smad7; right panel shows the quantitative analysis of acetylated Smad7/Smad7 ratio as measured by densitometry scanning of Western blots. Values are expressed in arbitrary units (a.u.). The blots are representative of three separate experiments in which LPMC of three normal controls were used. **(C,D)** Activation of Sirt1 in inflammatory bowel disease (IBD) LPMC reduces Smad7 acetylation and increases Smad7 ubiquitination. Total proteins extracted from IBD LPMC, cultured in the presence of CAY10591 or DMSO, were IP with Smad7 antibody or control antibody (neg ctr) and the blots were incubated with anti-acetyl-lysine antibody **(C)** or anti-ubiquitin antibody **(D)**. At the end, blots were stripped and incubated with a Smad7 antibody recognizing a different epitope of that recognized by the antibody used for immunoprecipitation. In panel **(C)**, arrow shows the specific band corresponding to acetylated Smad7; right panel shows the quantitative analysis of acetylated Smad7/Smad7 ratio as measured by densitometry scanning of Western blots. Values are expressed in arbitrary units (a.u.). The blots are representative of three separate experiments in which LPMC of three IBD patients were used. **(E)** Representative blots showing Smad7 and β-actin in total proteins extracted from IBD LPMC pre-incubated or not with proteasome inhibitors (Mg112 and Mg132) and then cultured in the presence of DMSO or Cay10591 for 8 h. The blots are representative of three separate experiments in which LPMC of three IBD patients were used.

Sirt1 deacetylates and hence promotes proteasomal-mediated degradation of various proteins (9). Since Smad7 stability and expression is mostly regulated by post-transcriptional mechanisms, which enhance Smad7 acetylation (7), we examined whether changes in Sirt1 activation following treatment of normal or IBD LPMC with Ex527 or Cay 10591, respectively, affected the status of Smad7 acetylation/ubiquitination. For this purpose, control and IBD LPMC were treated as indicated above, total proteins were immunoprecipitated with anti-Smad7 antibody, and the blots were incubated with either anti-ubiquitin antibody or anti-acetyl-lysine antibody. Inhibition of Sirt1 activity in Ex527-treated control LPMC enhanced Smad7 acetylation (Figure 4A) and markedly reduced Smad7 ubiquitination (Figure 4B). By contrast, treatment of IBD LPMC with Cay 10591 reduced Smad7 acetylation (Figure 4C) and enhanced ubiquitination (Figure 4D). To prove that the diminished Smad7 expression seen in Cay 10591-treated IBD LPMC was dependent on the ubiquitination-mediated proteasomal degradation, IBD LPMC were pre-incubated with proteasome inhibitors and then treated with Cay 10591. Cay 10591-driven Smad7 protein expression reduction was fully preventable by proteasome inhibition (Figure 4E).

### Smad7 Is a Negative Regulator of Sirt1

**Figure 5:**
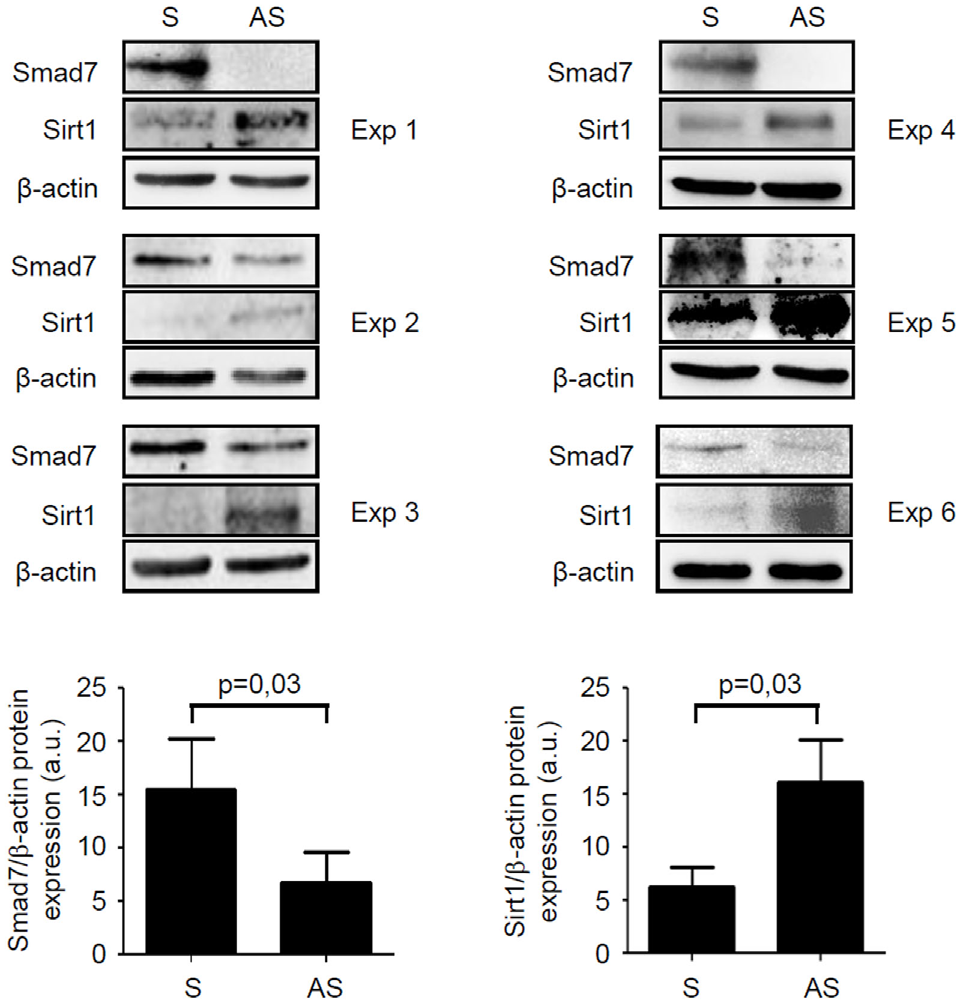
Knockdown of Smad7 increases Sirt1 expression in inflammatory bowel disease (IBD) mucosal explants. Blots showing Smad7, Sirt1, and β-actin in total proteins extracted from mucosal biopsy samples of six IBD patients treated with Smad7 sense (S) or antisense (AS) oligonucleotide. Results of each experiment (Exp) are shown. Lower panels show the quantitative analysis of Smad7/β-actin ratio and Sirt1/β-actin ratio, respectively, in six IBD patients as measured by densitometry scanning of Western blots. Values are expressed in arbitrary units (a.u.) and indicate mean ± SEM of all samples.

**Figure 6:**
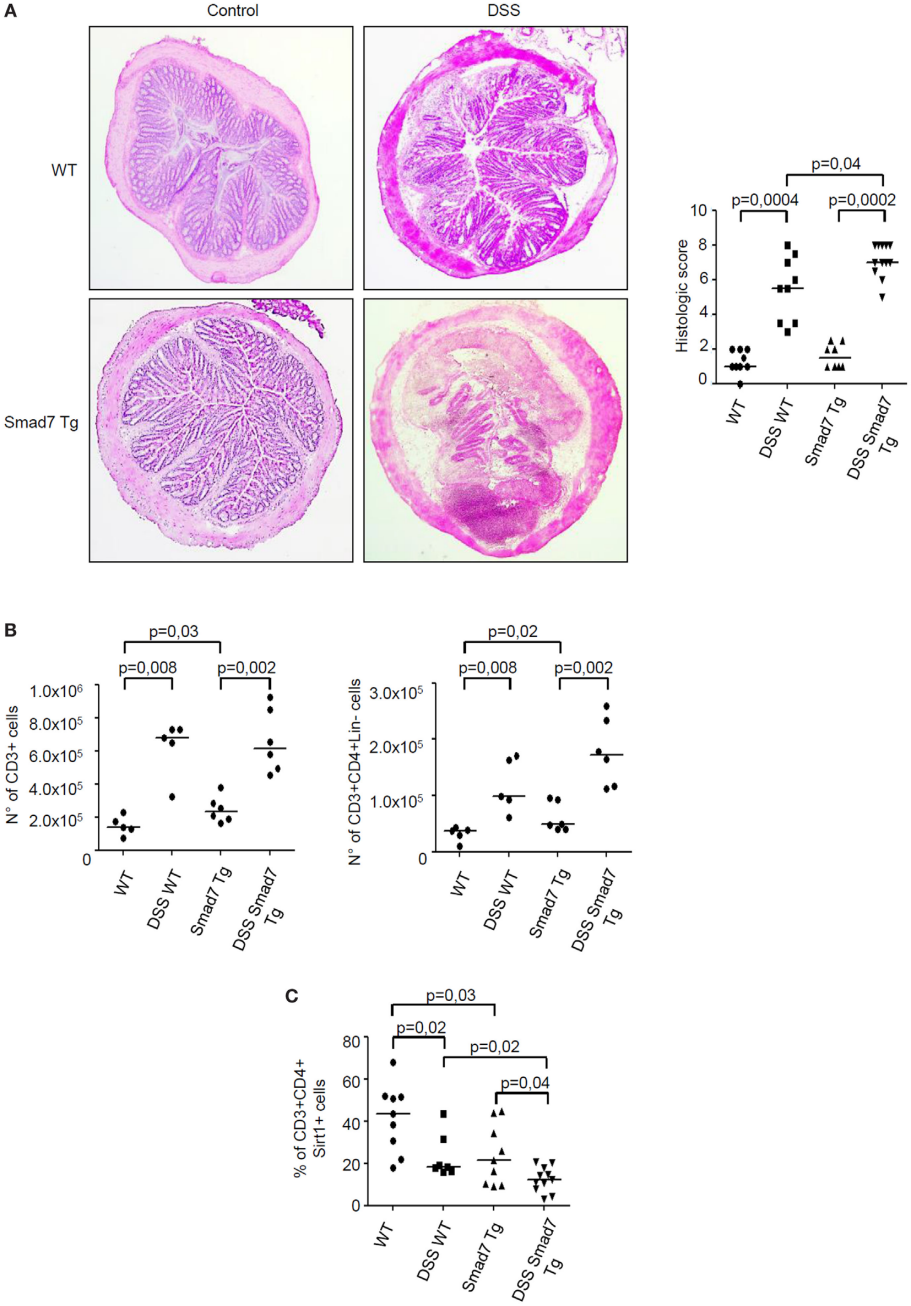
Sirt1 expression is reduced in the colon of mice overexpressing Smad7 in T cells. **(A)** Photomicrographs (50× original magnification) of hematoxylin and eosin-stained colonic sections taken from wild-type (WT) mice and Smad7 transgenic (Tg) mice treated or not treated with dextran sulfate sodium (DSS). Right panel shows histological score of the colon sections taken from WT and Tg mice either treated or not with DSS; each point in the graph indicates the histological score in the colon of a single mouse; horizontal bars indicate the median values. **(B)** Number of CD3+ cells (left panel) and CD3+CD4+Lin− cells (right panel) in the colons of mice treated as above. Lamina propria mononuclear cells (LPMC) were isolated from colonic samples, counted, and analyzed by flow cytometry. Each point in the graph indicates the number of positive cells in the colon of a single mouse; horizontal bars indicate the median values **(C)**. Percentage of Sirt1-expressing CD3+CD4+ LPMC isolated from colonic samples of mice treated as above and analyzed by flow cytometry; horizontal bars indicate the median values.

We have previously shown that inflammatory cytokines inhibit Sirt1 expression in the gut (10). Since high Smad7 contributes to amplify inflammatory signals in experimental models of colitis and IBD mucosa (5, 6, 14), we tested the possibility that Smad7 can negatively regulate Sirt1 expression. To address this issue, mucosal explants of IBD patients were treated with Smad7 AS and Sirt1 protein expression was then evaluated by Western blotting. Knockdown of Smad7 significantly enhanced Sirt1 protein expression (Figure 5). To confirm such data, we used a C57BL/6 CD2-Smad7 Tg mouse, which over-expresses Smad7 in T and NKT cells (12). Smad7 Tg mice did not spontaneously develop colitis. Induction of DSS-colitis was accompanied by a significant body weight loss in both WT and Smad7 Tg mice, with no significant difference between the two groups of animals (Figure S1A in Supplementary Material). However, histological analysis of colonic sections revealed that Smad7 Tg mice developed a more severe colitis in comparison to WT mice (Figure 6A). Moreover, inflamed colonic samples of Smad7 Tg mice had a more pronounced RNA expression for inflammatory markers, such as LCN2, IL-6, TNF-α, and IFN-γ when compared with inflamed samples of WT mice (Figure S1B in Supplementary Material). Flow cytometry analysis of mucosal LPMC revealed that, in basal conditions, Smad7 Tg mice had higher number of CD3+ and CD4+ cells than WT mice (Figure 6B). DSS treatment increased the number of CD3+ and CD3+CD4+ cells in both WT and Smad7 Tg mice without a significant difference between these two groups of mice (Figure 6B). Evaluation of Sirt1 expression in CD3+ and CD4+ cells showed that DSS treatment reduced the expression of Sirt1 in both WT and Smad7 Tg mice. Notably, Smad7 Tg mice had reduced levels of Sirt1 when compared with WT mice and this was evident in both unstimulated conditions and after DSS treatment (Figure 6C).

## Discussion

Accumulating evidence suggests that defects in counter-regulatory factors/mechanisms contribute to amplify inflammatory pathways in IBD (1, 2). In this context, we have previously shown that, in IBD mucosa, there are diminished activity of TGF-β1 due to elevated levels of Smad7, an intracellular inhibitor of TGF-β1 signaling (5), and reduced content of Sirt1, a deacetylase that controls negatively expression of various intracellular proteins (10). This study was undertaken to examine whether, in the gut, there is a reciprocal regulation between Smad7 and Sirt1 expression. Data of this work support and expand on our previous studies (5, 10) showing that, in IBD, both epithelial cells and LPMC express high Smad7 and low Sirt1 expression and activity. Smad7 can undergo two different modifications, namely, ubiquitination and acetylation, which both target the same lysine residues, with the downstream effect of differently regulating Smad7 protein content (7, 15). We here show that enhancing Sirt1 activity in IBD LPMC with a pharmacological compound reduces Smad7 acetylation, thereby promoting ubiquitination-driven proteasomal-mediated degradation of Smad7. On the other hand, inhibition of Sirt1 activity in normal LPMC enhances Smad7 expression. Altogether, these findings support the notion that Sirt1 can interact with various intracellular proteins (e.g., p53, forkhead family proteins) and modulate their functions through the deacetylation (11). Our data are in line with a previous study showing that Sirt1 modulates TGFβ-induced apoptosis in renal glomerular mesangial cells through direct interaction with Smad7 (16). Although we do not provide evidence of a direct control of Sirt1 on Smad7 in single cell types, it has been previously shown that Sirt1 directly interacts with and deacetylates Smad7 and accelerates the Smurf1-mediated ubiquitination and degradation of Smad7, thereby causing the instability of the protein (16). Since intestinal LPMC preparations contain different cell types (e.g., T cells, B cells, plasma cells, eosinophils, mast cells, antigen-presenting cells, stromal cells, and endothelial cells), further work is needed to ascertain in which cell types Sirt1 regulates Smad7 expression. Similarly, it remains to establish whether, in cells overexpressing p300, a protein with intrinsic acetyltransferase activity enhancing Smad7 stability (7), Sirt1 activation can eventually cause Smad7 protein downregulation.

These data suggest also that the reduced expression of Sirt1 in IBD mucosa relies in part on Smad7, as knockdown of Smad7 with a specific AS oligonucleotide enhances Sirt1 content in IBD mucosal explants. Some observations indicate that such a finding is not due to a class-related effect of the AS. We had previously shown that the anti-inflammatory effect of Smad7 AS oligonucleotide in IBD mucosal explants was reverted by a neutralizing TGF-β1 antibody (5). Moreover, in this study, we provide evidence that, in mice, Smad7-overexpressing T cells have low content of Sirt1 even in the absence of overt mucosal inflammation, supporting the hypothesis that Smad7 negatively regulates Sirt1 expression. Induction of DSS-colitis associates with significant reduction of Sirt1 in both WT and Smad7 Tg mice, but this effect is more prominent in Smad7 Tg mice. We would like to point out that these findings do not exclude the possibility that additional factors other than Smad7 contribute to reduce the mucosal levels of Sirt1 during colitis. In this context, we have previously shown that TNF and IL-21, two cytokines that are over-produced in IBD mucosa and do not affect Smad7 expression, downregulate Sirt1 in intestinal mucosal cells (10).

Although Smad7 Tg mice do not spontaneously develop colitis, flow cytometry analysis of LPMC preparations showed that over-expression of Smad7 increases accumulation of CD3+ and CD4+ T cells in the intestinal lamina propria, perhaps reflecting the Smad7-mediated loss of the inhibitory effect of TGF-β1 on T cell proliferation and activation (17, 18). Following DSS administration, Smad7 Tg mice develop a more severe colitis and exhibit a further reduction of the number of Sirt1-positive T cells when compared with WT mice. These results, together with our previous work showing that both Smad7 knockdown and activation of Sirt1 attenuate intestinal inflammation (5, 10), help delineate a scenario in which, in IBD mucosa, high Smad7 contributes to reduce Sirt1 content with the downstream effect of attenuating negative effects on its own expression and amplifying tissue-damaging immune responses. If this is the case, it is tempting to speculate that combined therapy with Smad7 AS, which suppresses the *de novo* induction of Smad7 protein, and Sirt1 activators, which promote degradation of Smad7 protein accumulated in mucosal cells other than exerting additional anti-inflammatory effects, could be more advantageous than monotherapy with single compounds.

## Ethics Statement

*Human subjects*: Each patient who took part in the study gave written informed consent. The study was approved by the Independent Ethic Committee at the Policlinico Tor Vergata of Rome (Rome, Italy) (experimentation register no. 42/12). *Animal subjects*: The study was approved by the Ministry of Health (authorization no. 324/2016-PR).

## Author Contributions

SS and GM participated in concept and research design, analyzed data, and wrote the manuscript. SS, EF, GB, MDG, and AR performed *in vitro* and *in vivo* experiments. AC and AO performed immunohistochemistry. EG and MG performed endoscopy and collected biopsy samples. GS collected and provided human specimens. All the authors agreed to be accountable for all aspects of this work.

## Conflict of Interest Statement

GM has filed a patent related to the treatment of inflammatory bowel diseases with Smad7 antisense oligonucleotides. The remaining authors declare no conflict of interest.

## Supplementary Material

Figure S1 Smad7 Tg mice develop more severe colitis following dextran sulfate sodium administration. **(A)** Change in body weight of Smad7 transgenic (Tg) mice and wild-type (WT) mice following dextran sulfate sodium (DSS) administration. **p* < 0.03, WT vs WT + DSS; Smad7Tg vs Smad7 Tg + DSS. **(B)** Induction of colitis by DSS associates with enhanced expression of RNA transcripts for inflammatory markers. Moreover, following DSS administration, Smad7-Tg mice exhibit a more pronounced expression of RNA transcripts for LCN2, IL-6, TNF-α, and INF-γ when compared with WT mice. Colonic samples were taken from eight mice per each group, analyzed by real-time PCR, and levels were normalized to β-actin. Data indicate mean ± SEM of all samples.
