# Supplementary figures and images for: Reciprocal Regulation Between Smad7 and Sirt1 in the Gut
Source: Front Immunol. 2018 Aug 10;9:1854. doi: 10.3389/fimmu.2018.01854 (PMC6097015; doi:10.3389/fimmu.2018.01854)

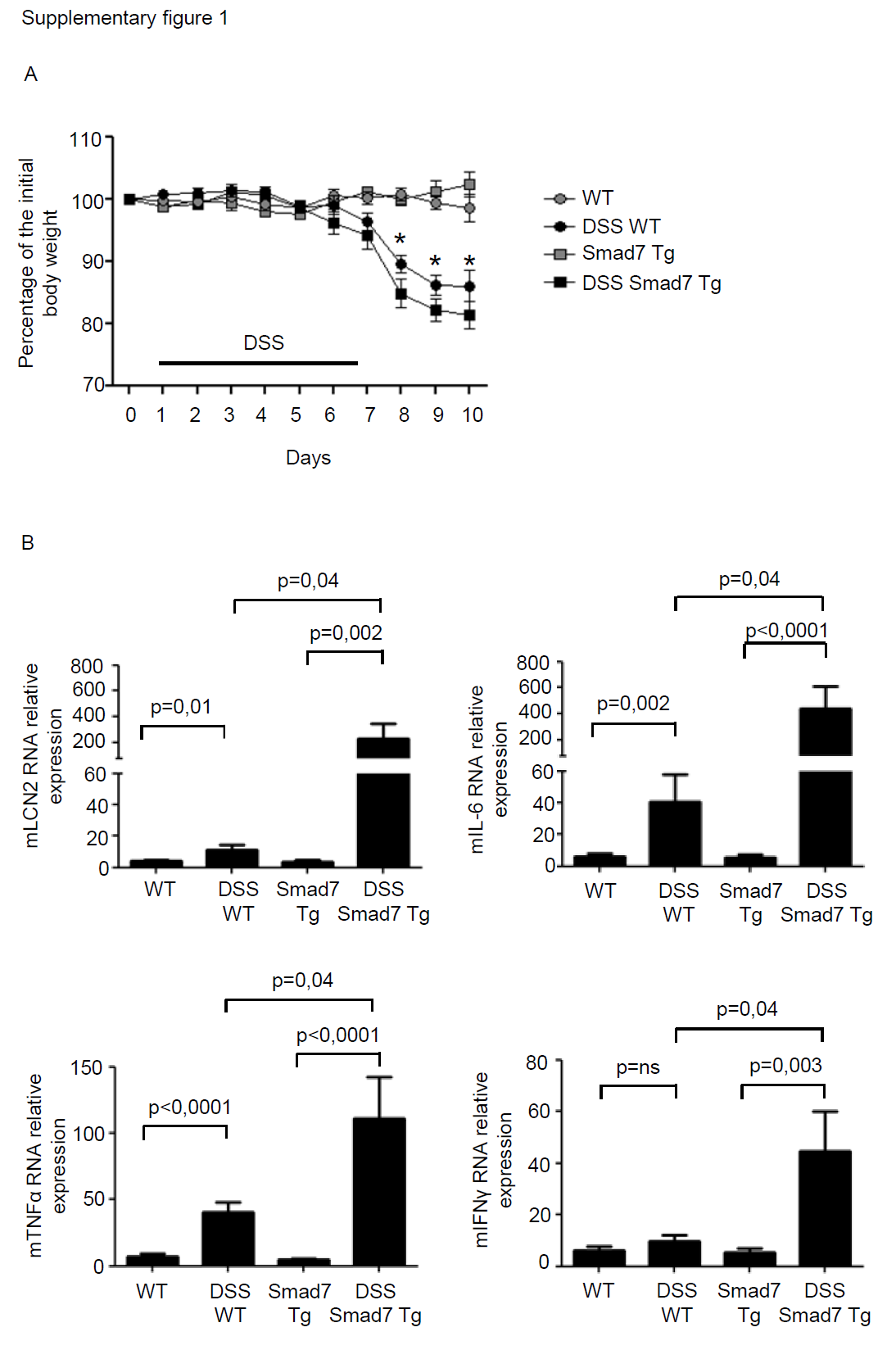

Supplement: Figure S1 — Smad7 Tg mice develop more severe colitis following dextran sulfate sodium administration. (A) Change in body weight of Smad7 transgenic (Tg) mice and wild-type (WT) mice following dextran sulfate sodium (DSS) administration. *p < 0.03, WT vs WT + DSS; Smad7Tg vs Smad7 Tg + DSS. (B) Induction of colitis by DSS associates with enhanced expression of RNA transcripts for inflammatory markers. Moreover, following DSS administration, Smad7-Tg mice exhibit a more pronounced expression of RNA transcripts for LCN2, IL-6, TNF-α, and INF-γ when compared with WT mice. Colonic samples were taken from eight mice per each group, analyzed by real-time PCR, and levels were normalized to β-actin. Data indicate mean ± SEM of all samples. [file Image_1.TIF]
